# Misdiagnosed esophageal perforation treated with endoscopic stent placement: a case report

**DOI:** 10.1186/1757-1626-2-6621

**Published:** 2009-05-14

**Authors:** Giuseppe R Nigri, Valentina Giaccaglia, Francesca Pezzoli, Paolo Aurello, Francesco D'Angelo, Emilio Di Giulio, Paolo Mercantini, Giovanni Ramacciato

**Affiliations:** Department of Surgery, Sapienza University of Rome2nd School of Medicine, St. Andrea Hospital, RomeItaly

## Abstract

Esophageal perforation has a high rate of mortality. Many strategies have been advocated for its management. Therapeutic options are surgical repair or resection, endoscopic placement of self-expandable metallic stents or, in selected cases, conservative management.

We describe a case of a 75-year-old man admitted to our hospital for forceful vomiting since 24 hours. The patient was treated with endoscopic placement of a covered self expandable metallic stent. Although the late diagnosis delayed the treatment, the patient survived the usually fatal condition. The stent removal was performed 8 weeks after implantation.

Immediate and correct diagnosis are the key elements in improving survival of patients with esophageal perforation. This has to be associated to the selection of the most appropriate treatment. Implantation of covered self-expandable metallic stents in compromised patients with esophageal perforation is a safe and feasible alternative to operative treatment.

## Introduction

Postemetic spontaneous esophageal perforation is named after Herman Boerhaave who, in 1723, described the death of Dutch Grand Admiral, Baron Jan van Wassenaer, caused by esophageal rupture after a heavy meal, following forceful vomiting [1]. Spontaneous esophageal rupture is a surgical emergency with a high mortality rate (>90%) [2], its diagnosis can be difficult as the presentation is often non specific and can be confused with other disorders such as perforated peptic ulcer, pancreatitis, myocardial infarction, pneumonia, spontaneous pneumothorax or dissecting aortic aneurysm [3].

The chances of survival increase with prompt diagnosis and early treatment, the latter involving either surgical or non-operative treatment [4-7].

We report the case of a patient with spontaneous esophageal rupture to highlight the importance of early recognition and treatment and the criteria, which guided our choice of the most appropriate management.

## Case presentation

We present the case of a 75-year-old Caucasian man admitted to our hospital for forceful vomiting since 24 hours, dyspnea and progressively worsening abdominal pain. His past medical history was positive for occlusive arteriopathy of the lower extremities; for this reason 6 months before he underwent to right femoro-femoral bypass and immediately after to Fogarty embolectomy for occlusion of the bypass graft.

When the patient was admitted to the hospital, he was in bad clinical conditions, with no fever, and his oxygen saturation measured by pulse oximetry (SpO2) was 90% on 40% oxygen via Ventimask at 8 l/min. The laboratory values were as follows: white blood cell count, 24.600/ml; red blood cell count, 308x104/ml; haemoglobin, 11.9 g/dl; CRP, 9.93mg/dl. A chest radiograph and abdominal ultrasound showed no abnormalities.

The patient was then moved to another unit for observation. Two days after being admitted to the hospital, following a vomiting episode and worsening of patient's clinical picture, who was now febrile and strongly tachypnoic (30 R/min) and dyspnoeic (SaO2 78% in VM 40% and 8 l/min), a chest CT was performed. The CT showed a massive left hydropneumothorax with full collapse of the homolateral lung and counter-lateral deviation of the mediastinum. Two chest drains were then positioned in the left hemithorax.

In the days that followed, we witnessed the continuous worsening of patient's clinical conditions, with anemia (Hb: 7.4 g/dl), worsening of kidney functionality, increase of PCR (21.08 mg/dl) and continuous abundant outflow (1000 ml) from chest drains of purulent material mixed with blood. The chemical, physical and bacteriological analysis of the pleural liquid proved positive to *Klebsiella pneumoniae*, *Enterobacter cloacae* and *candida tropicalis*. When consulted, we suggested the opportunity of performing a chest-abdomen CT with Gastrografin: the examination showed the spreading of the contrast agent in the left pleural cavity and continuous solution in the distal third of the oesophagus. The patient was then moved to our unit and diagnosed the rupture of the distal oesophagus with left esophageal-pleural fistula. The patient was then fasted and fed exclusively with TPN, associated with a targeted antibiotic therapy. Considering the general bad conditions, it was decided not to surgically treat the esophageal perforation and to place an endoluminal esophageal stenting by endoscopy. A self expansible covered metallic stent (Deltamed, Italy) was placed. The following X-rays series with gastrografin did not show seamless flows in the esophageal wall. In the days that followed, we witnessed the continuous improving of patient's clinical conditions, as he progressively resumed oral nutrition. The patient was discharged 10 days after the transfer in fairly good general conditions and able to eat autonomously. The stent was removed after 2 months and the patient is alive at 8 months.

The causes of esophageal rupture are various and are classified into three types: iatrogenic, traumatic and spontaneous. Twenty to 40% of all cases are spontaneous [2]. Spontaneous esophageal perforation is extremely dangerous and occasionally fatal condition because it may rapidly progress to severe mediastinitis, sepsis and multiple organ failure.

The time from esophageal perforation until effective therapy is started is of paramount importance and is the key to a successful outcome in all cases we analyzed [5,6,8].

The patient, in a small percentage of cases, may present with Mackler's classic triad (vomiting, chest pain and subcutaneous emphysema) or, mostly, with atypical symptoms [5]. In particular, our case had vomiting and chest pain but not subcutaneous emphysema.

There are some imaging techniques that can support diagnosis, such as: Chest X-rays (which can show a pneumomediastinum and a pleural effusion often localized to the left), EGDS, esophagogram with Gastrografin and CT with contrast agent [4]. In our case, the initial study of the patient with chest X-ray and abdominal ultrasonography did not show any sign suggesting esophageal perforation. The chest CT performed a few days after showed such a very severe lung picture (massive hydropneumothorax with counter-lateral deviation of the mediastinum) which suggested therapeutic priority and highlighted a diagnostic latency that led to the important worsening of patient's general conditions. What proved to be crucial in the diagnosis of the correct clinical picture of the patient was a chest-abdomen CT with contrast agent by oral administration, which was able to highlight the fistula opening between the oesophagus and the left pleural cavity.

After the esophageal perforation was diagnosed, the most critical decision was to choose the most appropriate therapeutic solution. As of today, choosing a surgical approach is considered as the gold standard, since it provides direct closing of the esophageal opening and draining of the affected cavities [9]. Two commonly used sugical strategies for treatment of transmural intrathoracic esophageal perforations are primary repair and an esophageal diversion. Both these strategies include major surgery with thoracotomy, which may be hazardous to perform in elderly patients and patients with concomitant diseases. In patients where the surgical option involves some risk, as in severely ill patient, septic or with important concomitant diseases, a conservative treatment that is introducing stents that can restore the esophageal continuity - offers interesting results [5,8,10]. In our case, the bad clinical conditions and the presence of an important vasculopathy, suggested to try a conservative approach through endoscopic introduction of a metal self-expandable stent.

## Conclusions

In conclusion, our case suggests always to consider a possible esophageal rupture in those patients with chest or gastric pain, even after one single vomiting episode, in order to provide a correct diagnosis allowing a targeted treatment. A conservative treatment of an esophageal perforation can guarantee excellent results, in particular in severely ill patients where surgery would be associated with a high mortality rate.

## List of abbreviations

CT: Computerized tomography
EGDS: esophagogastroduodenoscopy
PCR: Polymerase chain reaction
